# Graphene-Based Polymer Nanocomposites: Recent Advances

**DOI:** 10.3390/polym14102102

**Published:** 2022-05-21

**Authors:** Ana M. Díez-Pascual

**Affiliations:** Universidad de Alcalá, Facultad de Ciencias, Departamento de Química Analítica, Química Física e Ingeniería Química, Ctra. Madrid-Barcelona, Km. 33.6, 28805 Alcalá de Henares, Madrid, Spain; am.diez@uah.es; Tel.: +34-918-856-430

Carbon-based nanomaterials are currently attracting a great deal of interest due to their unique chemical, optical, and electronic properties, which make them suitable for a broad range of uses, including supercapacitors, solar cells, fuel cells, lithium batteries, biomedicine, and so forth. Among them, graphene and its related materials (GRMs), namely graphene oxide (GO) and reduced graphene oxide (rGO), have been widely investigated (Figure 1). GO is the oxidized form of graphene and can be prepared via oxidation and exfoliation of graphite. rGO can be prepared from GO via chemical, thermal or electrochemical reduction. The addition of these GRMs to polymers (i.e., thermoplastics, epoxies, conducting polymers, biopolymers, etc.) can lead to functional nanocomposites with significantly improved properties due to synergistic effects [1,2].

Thus, the properties of GRM/polymer nanocomposites can be simply tuned by cautiously adjusting the GRM concentration, its synthesis path and the GRM-polymer interaction. In this concern, covalent and non-covalent functionalization routes have been applied to finely tune the GRM surface with the goal of boosting its dispersion and interfacial interaction with the host matrix [3,4]. The non-covalent tactics rely on physical interactions, such as physical adsorption and/or wrapping on the GRM surface [5]. Three main techniques have been reported to prepare GRM-based nanocomposites: solution mixing, melt-blending, and in situ polymerization. The first approach requires the choice of a proper solvent in which both the GRM and the matrix are dissolved [6,7,8,9]; it results in a good mixing between the exfoliated graphitic flakes and polymer matrix. However, graphene has a strong tendency to aggregation and shows poor solubility, which are the dominant factors for limiting the application of this technique. The second comprises the blending of the GRM with a molten polymer matrix under strong shear forces [10]. This approach is environmentally benign due to the absence of organic solvents and industrially attractive due to its scalability and low-cost, but is typically limited to thermoplastic matrices. The third implies the polymerization of monomers in the presence of the GRM, process initiated by light irradiation or heat [11], and is the most widely used to prepare GRM composites with conjugated polymers. The covalent method relies on the formation of a chemical bond between the GRM and the polymer [12], resulting in a strong interfacial interaction, nonetheless can disturb the conjugated п system of the GRM, hence altering its properties. 

This Special Issue provides selected examples of the most recent advances in the preparation and characterization of polymer nanocomposites incorporating graphene or its derivatives for a variety of applications.

Numerous biosensors based on GRMs have been recently developed [13]. For instance, biosensor-based detection of acetone vapor, a good biomarker for the non-invasive diagnosis of diabetes, has been recently reported using a ternary composite comprising p-toluene sulfonic acid (PTSA) doped polyaniline (PANI), chitosan and rGO [14]. It consists in an optical biosensor based on surface plasmon resonance (SPR) technique. The ternary composite film was spin-coated, and its structure, morphology and chemical composition were characterized by FTIR, UV-VIS, FESEM, EDX, AFM, XPS, and TGA. The ternary composite-based SPR sensor had a limit of detection of acetone vapor of 0.88 ppb. The selectivity, repeatability, reversibility, and stability of the sensor were also studied. The acetone response was 87%, 94%, and 99% higher compared to common interfering volatile organic compounds, such as propanol, methanol, and ethanol, respectively. 

Recently, graphene and GO are becoming widely used in tissue engineering [15]. These can be applied in drugs and genes delivery of, as well as in the articular repair and bone defects [16,17], due to their physical characteristics such as mechanical strength, flexibility, elasticity, low density, structural support, self-lubricating properties and so forth. Furthermore, these can be applied to mediate cell proliferation, differentiation and migration and improve the bone repair effect. On the other hand, poly (L-lactic acid) (PLLA) is a biocompatible polymer widely used in the biomedical field, particularly in osteosynthesis bioengineering due to its greater commercial potential as a versatile biodegradable plastic, its thermoplastic processability, low price and good mechanical properties [18,19], and for its biodegradability and biocompatibility. These qualities added to its use in the device’s design for bone fixation and controlled drug release. In this regard, Santos Silva et al. [20] developed filaments of PLLA and GO nanocomposites, verifying the capacity of mesenchymal stem cells (MSCs) to adhere to the nanocomposites after 7 days in vitro. Using macroscopic analysis, GO implanted in the subcutaneous region, forming a structure similar to a ribbon, without tissue invasion. Histologically, no tissue architecture changes were observed, and PLLA-GO cell adhesion was demonstrated by scanning electron microscopy (SEM).

Improving the fire-retardant behavior of polymers is a major challenge for extending their use in most applications. The addition of graphene nanoplatelets has been reported a successful mean to improve the fire retardancy of polymeric composites. In this regard, fire-retardant systems consisting of GNPs and polyethylene terephthalate (PET) foam were developed in water solution [21]. Coating amounts of 1.5, 2.5, and 3.5 wt% were chosen for the preparation of the samples. Flammability, flame penetration, and combustion tests confirmed the enhancement of the foam properties via coating. The time to ignition increased and the peak of heat release rate was drastically reduced by up to 60% compared to that of the uncoated PET foam. Scanning electron microscopy (SEM) images proved the morphological effect of the heat treatment on the surface, showing that the coating was uniformly distributed. 

Nanocomposites based on conducting polymers and GRMs have attracted much attention for applications in chemical sensors, light-emitting diodes (LEDs), organic solar cells (OSCs), etc. GO is an ideal filler due to its unique properties to reinforce polymeric matrices. However, it needs to be functionalized to expand its solubility in common solvents and allow the processing by low-cost solution deposition methods. In a recent work [9], hexamethylene diisocyanate (HDI)-modified GO and its nanocomposites with poly(3,4-ethylenedioxythiophene): poly(styrenesulfonate) (PEDOT:PSS) were developed, and their morphology, thermal, electrical, thermoelectrical and mechanical performance were characterized. The influence of the HDI functionalization degree and concentration on the nanocomposite properties was evaluated. The HDI-GO increased the crystallinity, lamella stacking and interchain coupling of PEDOT:PSS chains. In addition, a strong improvement in electrical conductivity, thermal stability, Young’s modulus and tensile strength was found, showing an optimum combination at 2 wt% loading. Drop and spin casting techniques were applied onto different substrates, and the results from deposition tests were analyzed by atomic force microscopy (AFM) and UV–vis spectroscopy. This study opens new routes towards further research on conducting polymer/modified GRM nanocomposites to optimize their composition and properties for use in OSCs.

At present, plastic pollution is a severe threat facing humans, animals and plants. The development of bioplastic materials is vital to solve our global environmental challenges and to preserve the prosperity of our world. In this regard, bio-based plastics are pursued. Amongst the most promising bioplastics is polyhydroxybutyrate (PHB), which is a microbial polyester belonging to the polyhydroxyalkanoate (PHA) family. This biocompatible and non-toxic polymer is biosynthesised by bacterial fermentation from renewable resources [22,23]. It is highly crystalline due to its linear chain structure and also has a number of advantages over synthetic polymers for certain biomedical applications and the production of food packaging, including superior barrier performance to both polyethylene (PE) and polypropylene (PP). The main shortcomings of PHB, are its low thermal stability, poor impact resistance and lack of antibacterial activity. This issue can be improved by blending with other biodegradable polymers, such as polyhydroxyhexanoate to form poly(3-hydroxybutyrate-co-3-hydroxyhexanoate) (PHBHHx). However, PHBHHx shows reduced stiffness than PHB and poorer barrier properties. Recently, new biodegradable PHBHHx/GO nanocomposites were prepared via a simple, cheap and environmentally friendly solvent casting method. The morphology, mechanical, thermal, barrier and antibacterial properties of the nanocomposites were evaluated. The stiffness and strength of the biopolymer were enhanced up to 40% and 28%, respectively. Moreover, the nanocomposites showed superior thermal stability, lower water uptake and better gas and vapor barrier properties than neat PHBHHx. They also showed strong biocide action against Gram-positive and Gram-negative bacteria. These GRM-based nanocomposites can replace conventional petroleum-based synthetic polymers presently used for food packaging.

## Figures and Tables

**Figure 1:**
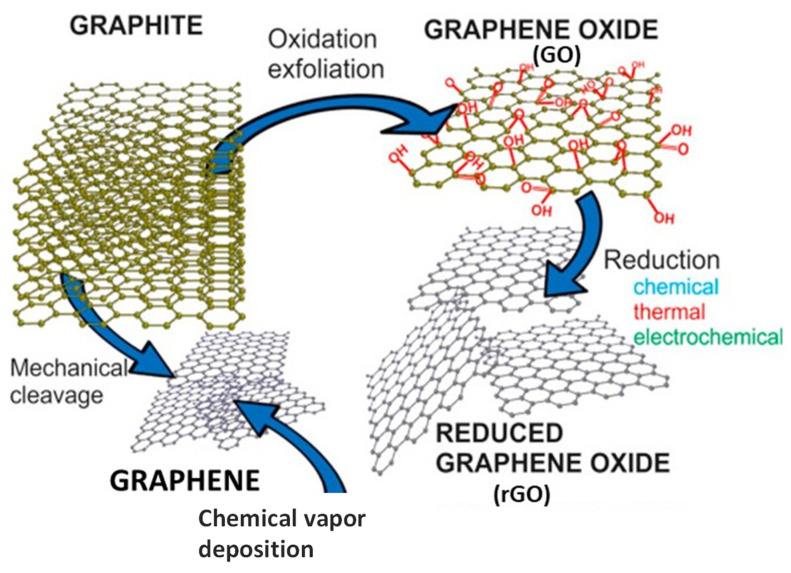
Schematic representation of graphene-related materials (GRMs) and their synthesis methods.
